# Supplementary material for: Baseline Quality of Life of Physical Function Is Highly Relevant for Overall Survival in Advanced Rectal Cancer
Source: Healthcare (Basel). 2022 Jan 12;10(1):141. doi: 10.3390/healthcare10010141 (PMC8775862; doi:10.3390/healthcare10010141)
Supplement: Supplementary file 1 [file healthcare-10-00141-s001.zip › healthcare-1517091-supplementary.pdf]

Supplementary “Patients and Methods”

Links to the different questionnaires and the manual:

Link to the English version of the QLQ-C30:

https://www.eortc.org/app/uploads/sites/2/2018/08/Specimen-QLQ-C30-English.pdf

Link to the English version of the QLQ-CR38:

https://qol.eortc.org/?s=qlq-cr38

Link to the questionnaires and the user agreement

https://qol.eortc.org/questionnaires/

Link to the manual for the use and evaluation of the EORTC questionnaires

https://www.eortc.org/app/uploads/sites/2/2018/02/SCmanual.pdf

The links were deposited on 30.12.2021.

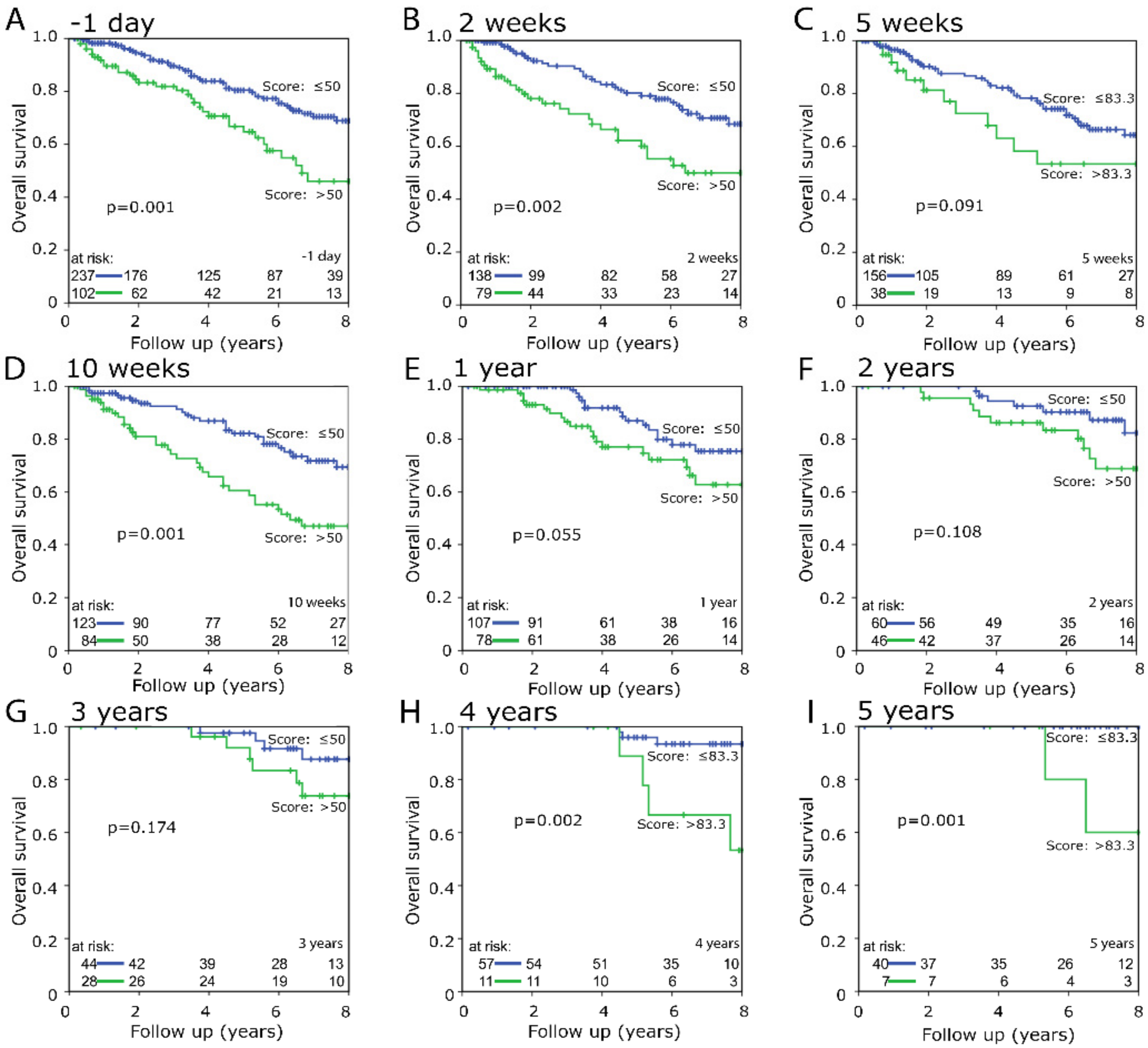

Supplement Figure S1: Overall survival between the group with higher (green line) and lower (blue line) scoring "strenuous activities" with respective cutoff values at the different dates (**A:** day -1, **B:** week 2, **C:** week 5, **D:** week 10, **E:** 1 year, **F:** 2 year, **G:** 3 year, **H:** 4 year, **I:** 5 year.

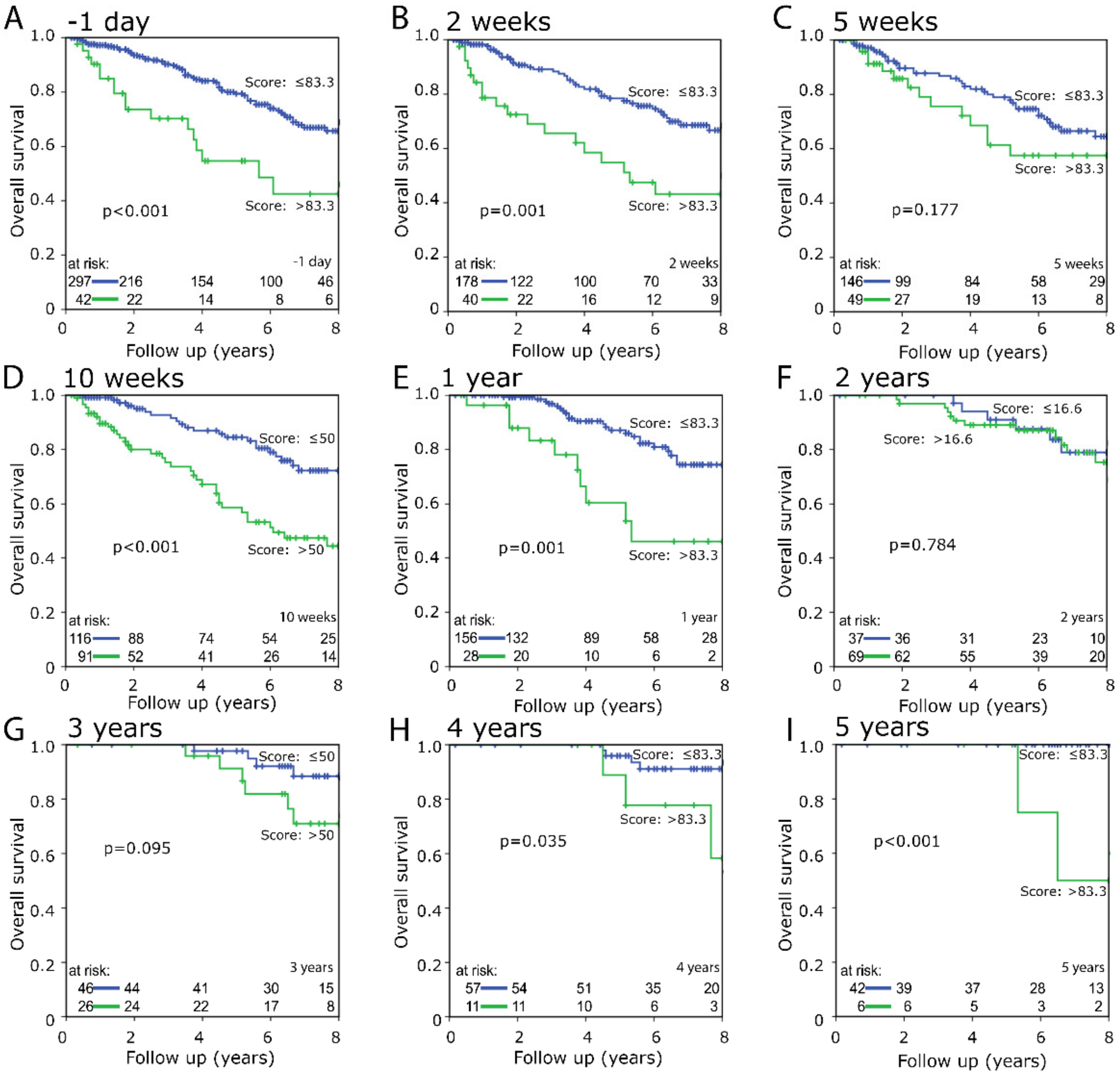

Supplement Figure S2: Overall survival between the group with higher (green line) and lower (blue line) scoring "long walk" with respective cutoff values at the different dates (**A:** day -1, **B:** week 2, **C:** week 5, **D:** week 10, **E:** 1 year, **F:** 2 year, **G:** 3 year, **H:** 4 year, **I:** 5 year.

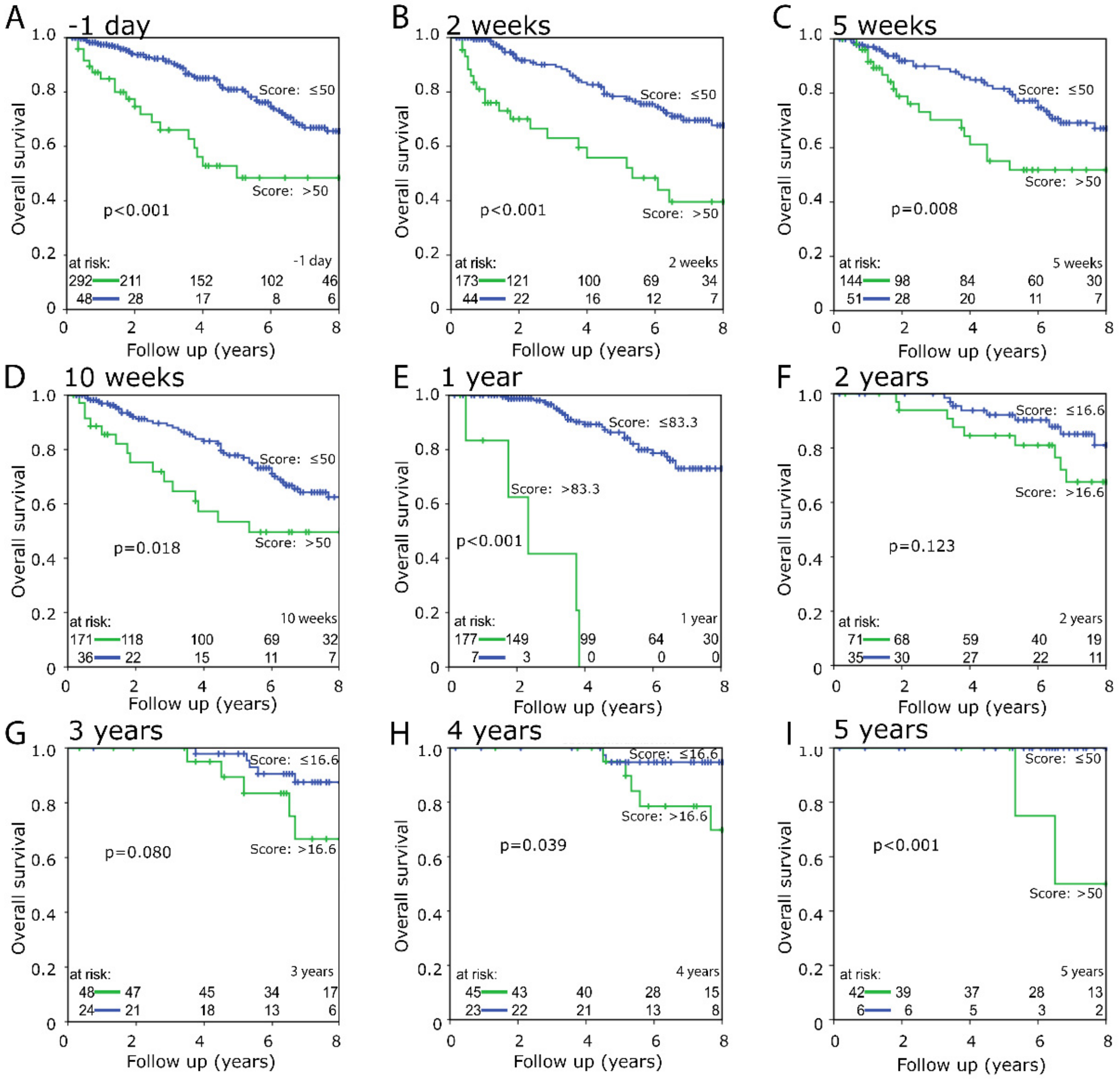

Supplement Figure S3: Overall survival between the group with higher (green line) and lower (blue line) scoring "short walks" with respective cutoff values at the different dates (**A:** day -1, **B:** week 2, **C:** week 5, **D:** week 10, **E:** 1 year, **F:** 2 year, **G:** 3 year, **H:** 4 year, **I:** 5 year.

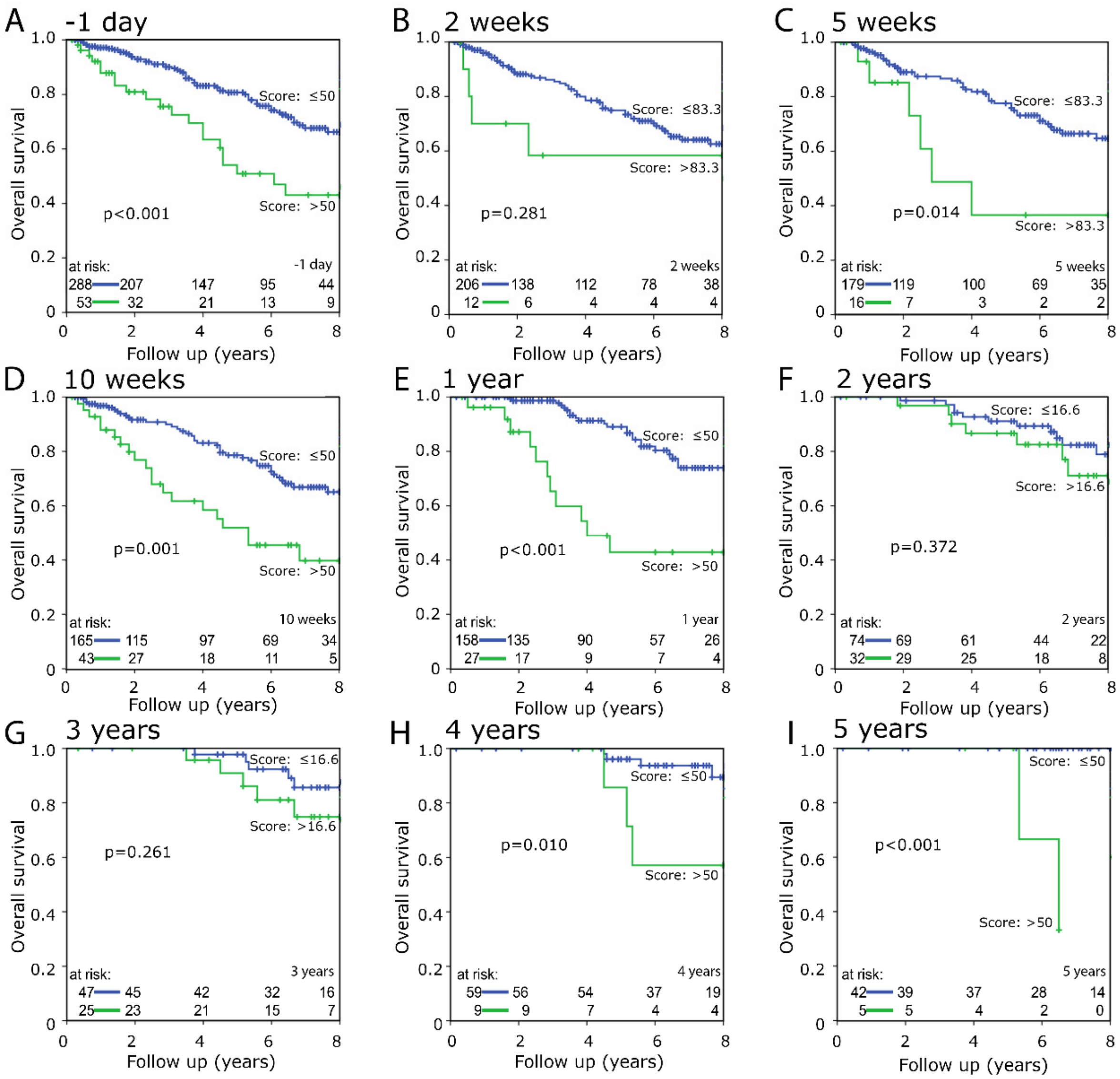

Supplement Figure S4: Overall survival between the group with higher (green line) and lower (blue line) scoring “bed and chair time” with respective cutoff values at the different dates (**A:** day -1, **B:** week 2, **C:** week 5, **D:** week 10, **E:** 1 year, **F:** 2 year, **G:** 3 year, **H:** 4 year, **I:** 5 year.

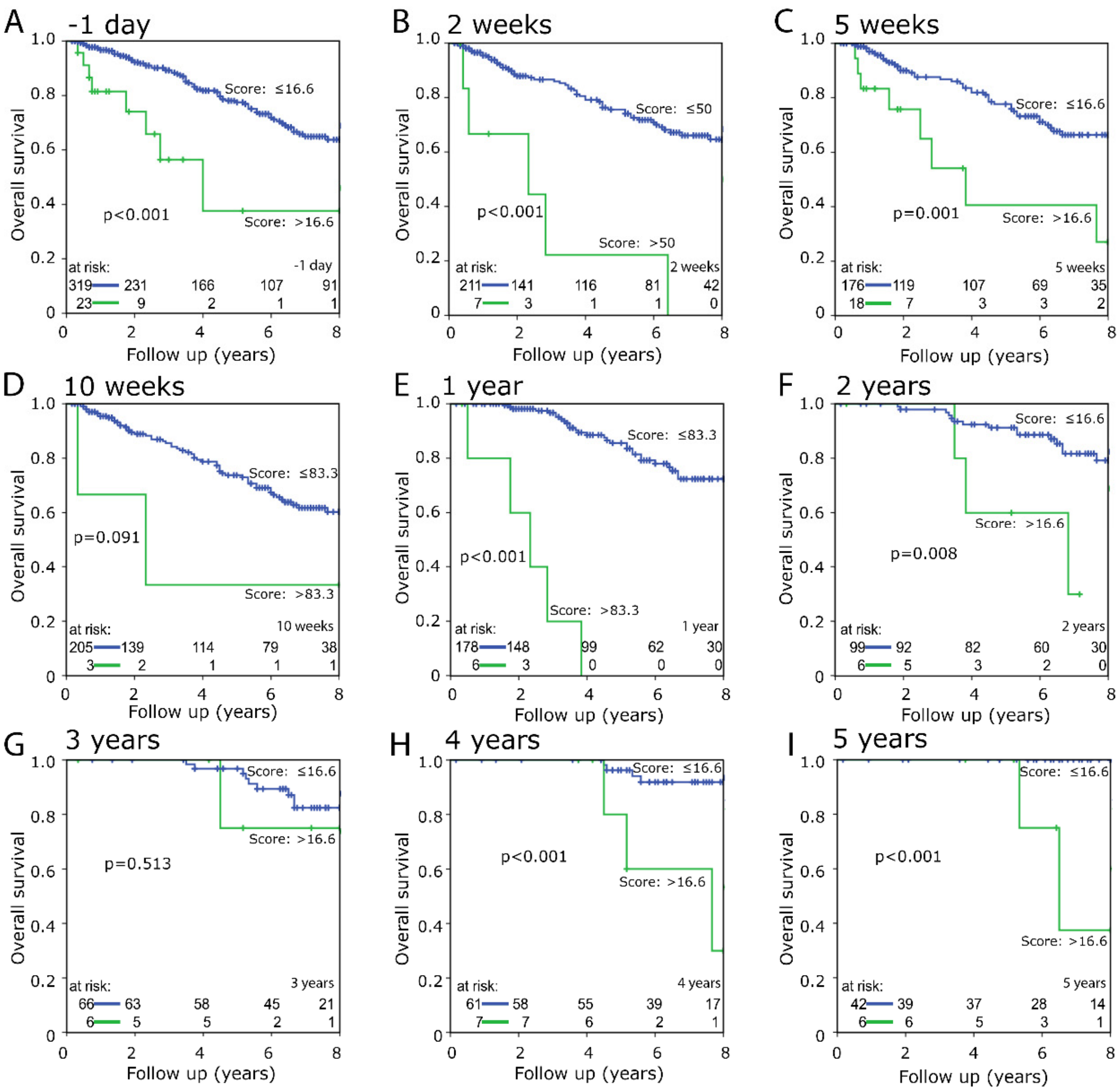

Supplement Figure S5: Overall survival between the group with higher (green line) and lower (blue line) scoring "daily help" with respective cutoff values at the different dates (**A:** day -1, **B:** week 2, **C:** week 5, **D:** week 10, **E:** 1 year, **F:** 2 year, **G:** 3 year, **H:** 4 year, **I:** 5 year.

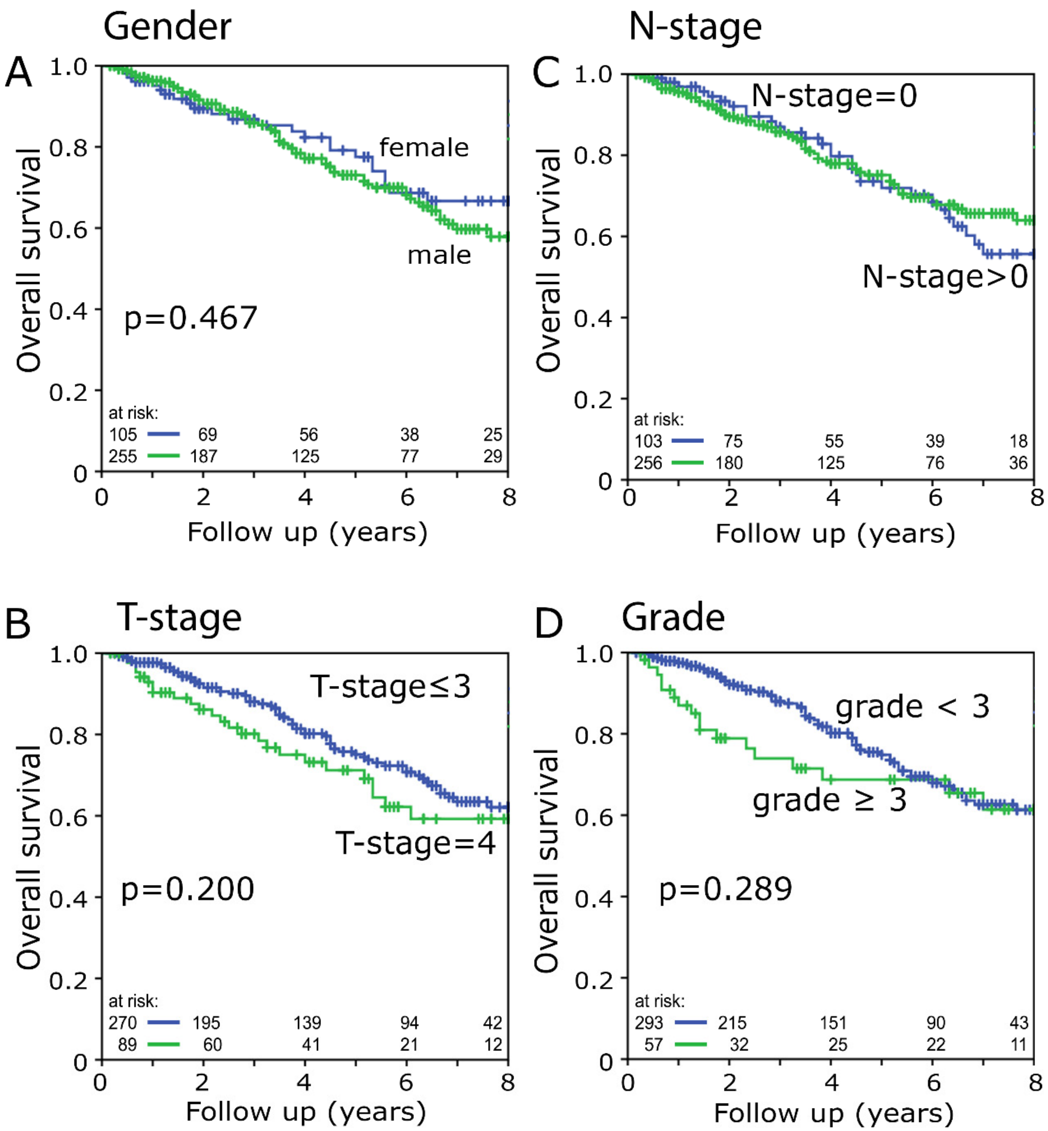

Supplement Figure S6: A Overall survival by gender, female (blue line) and male (green line); **B:** Overall survival by T stage, T≤3 (blue line) and T=4 (green line); **C:** Overall survival by nodal stage, N=0 (blue line) and N=1/2 (green line) **D:** Overall survival by grading, G<3 (blue line) and G≥3 (green line).

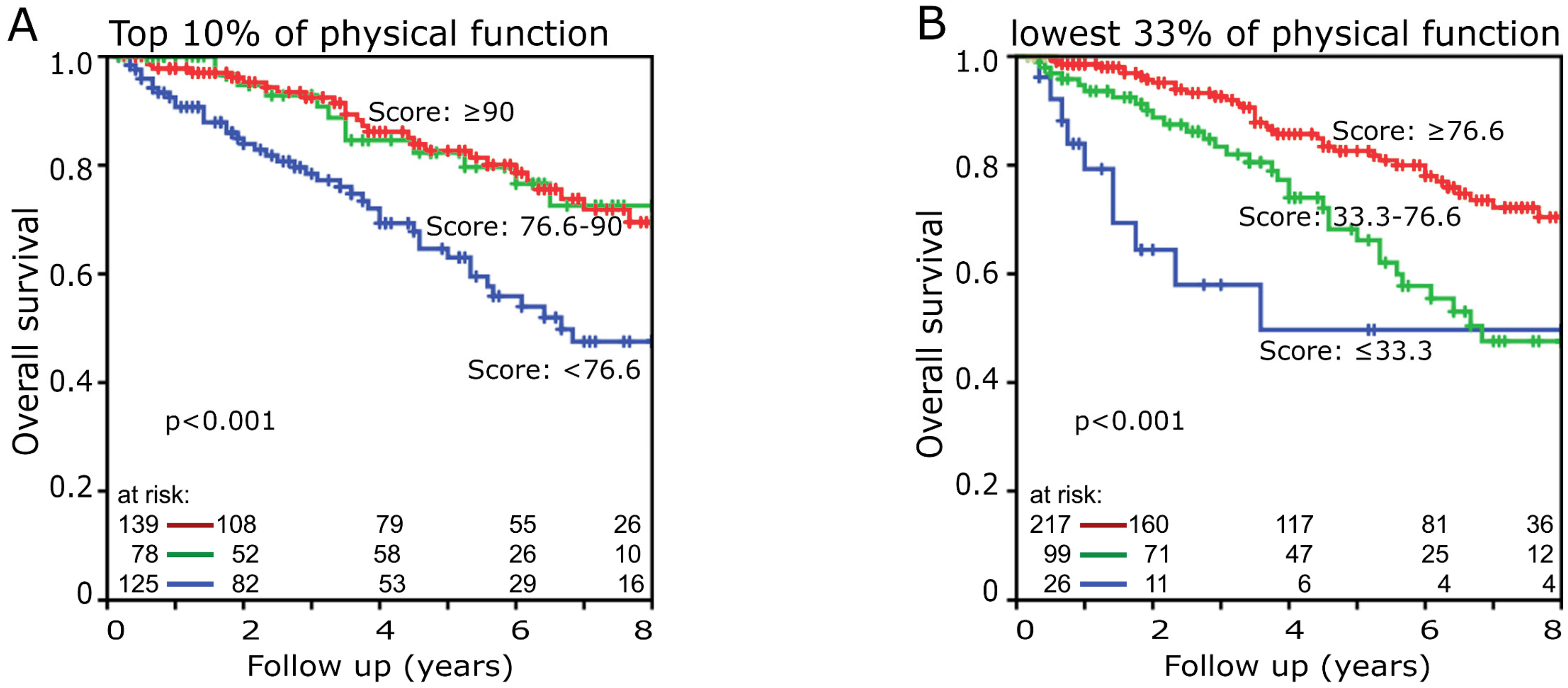

Supplement Figure S7: A Overall survival in 3 levels of "physical function" with a score from 90-100% (red line), 76.6-90% (green line) and below 76.6% (blue line); **B** Overall survival in 3 levels of "physical function" with a score of 76.6% and higher (red line), 33.3-76.6% (green line) and 33.3% and below (blue line).

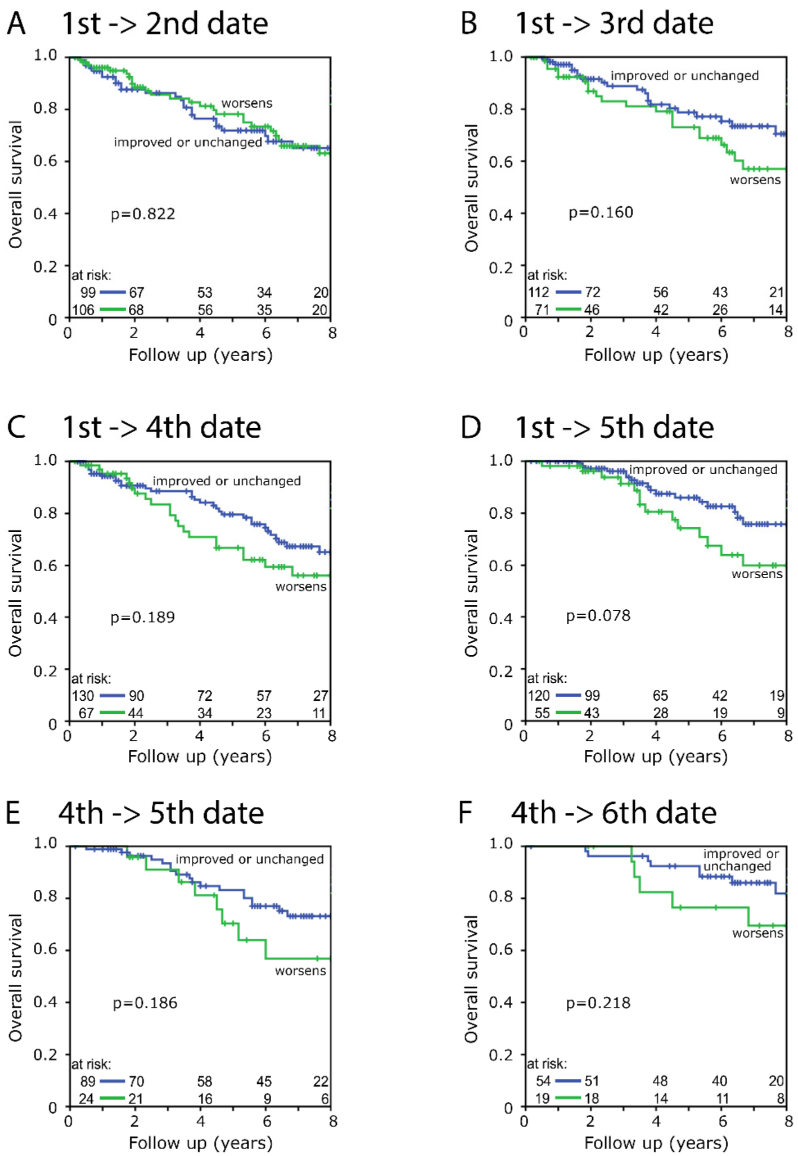

Supplement Figure S8: Overall survival by "physical function" score changes between various dates (**A** 1st to 2nd; **B** 1st to 3rd; **C** 1st to 4th; **D** 1st to 5th; **E** 4th to 5th and **F** 4th to 6th date).
